# 

**Published:** 1873-07

**Authors:** 


### CONTENTS:

### ORIGINAL COMMUNICATIONS.

ARTICLE I.—Kentucky and Indiana State Dental Associations ........ 97

Diagnosis in Practice. By Dr. D. C. Hauxhurst........ 102

The Dentist of the Future. By Dr. W. F. Morill........ 106

ARTICLE II.—Mechanical Dentistry. By Dr. A. W. French........ 111

ARTICLE III.—American Dental Convention. By J. G. Ambler, D. D. S........ 118

American Dental Association. By M. S. Dean, D. D. S........ 119

Southern Dental Association, Fifth Annual Meeting........ 133

### SELECTED ARTICLES.

ARTICLE IV.—Furrowed Enamel in Connection with Syphilitic Diseases. By S. P. Cutler, M. D., D. D. S. (Concluded.) ........ 120

ARTICLE V.—Dental Physiology. By H. S. Chase, M. D., D. D. S ........ 125

### EDITORIAL DEPARTMENT.

A Model Hospital........ 134

The Discoverer of Anæsthesia ........ 137

### BIBLIOGRAPHICAL.

A System of Dental Surgery. By John Tomes, F. R. S., and Chas. Tomes, M. A. Second Edition ........ 140

A Manual of Dental Mechanics. By Oakley Coales, L. R. C. S ........ 140

On Strictures of the Urethra. By F. N. Otes, M. D........ 141

### MONTHLY SUMMARY.

A Remedy for Hæmoptysis........ 141

Vehicle for Chloral Hydrate........ 142

Nervousness ........ 142

Oatmeal, Bone and Muscle........ 142

Suppression of Perspiration........ 143

Diarrhœa in Teething........ 143

Odor of Orris Root........ 144

Death During Etherization........ 144

